# Atomic switches of metallic point contacts by plasmonic heating

**DOI:** 10.1038/s41377-019-0144-z

**Published:** 2019-03-27

**Authors:** Weiqiang Zhang, Hongshuang Liu, Jinsheng Lu, Lifa Ni, Haitao Liu, Qiang Li, Min Qiu, Bingqian Xu, Takhee Lee, Zhikai Zhao, Xianghui Wang, Maoning Wang, Tao Wang, Andreas Offenhäusser, Dirk Mayer, Wang-Taek Hwang, Dong Xiang

**Affiliations:** 1 Tianjin Key Laboratory of Optoelectronic Sensor and Sensing Network Technology, Key Laboratory of Optical Information Science and Technology, Institute of Modern Optics, College of Electronic Information and Optical Engineering, Nankai University, 300350 Tianjin, China; 2 State Key Laboratory of Modern Optical Instrumentation, College of Optical Science and Engineering, Zhejiang University, 310027 Hangzhou, China; 3 College of Engineering, University of Georgia, Athens, GA 30602 USA; 4 Department of Physics and Astronomy, and Institute of Applied Physics, Seoul National University, Seoul, 08826 Korea; 5 Institute of Materials Research and Engineering, A*STAR, 2 Fusionopolis Way, Innovis, Singapore, 138634 Singapore; 6 Institute of Complex Systems, ICS-8, Bioelectronics, Research Center Juelich and JARA Fundamentals of Future Information Technology, Jülich, 52425 Germany

**Keywords:** Photonic devices, Atom optics, Nanophotonics and plasmonics

## Abstract

Electronic switches with nanoscale dimensions satisfy an urgent demand for further device miniaturization. A recent heavily investigated approach for nanoswitches is the use of molecular junctions that employ photochromic molecules that toggle between two distinct isoforms. In contrast to the reports on this approach, we demonstrate that the conductance switch behavior can be realized with only a bare metallic contact without any molecules under light illumination. We demonstrate that the conductance of bare metallic quantum contacts can be reversibly switched over eight orders of magnitude, which substantially exceeds the performance of molecular switches. After the switch process, the gap size between two electrodes can be precisely adjusted with subangstrom accuracy by controlling the light intensity or polarization. Supported by simulations, we reveal a more general and straightforward mechanism for nanoswitching behavior, i.e., atomic switches can be realized by the expansion of nanoelectrodes due to plasmonic heating.

## Plasmonics: Tripping the light fantastic switch

Scientists have shown how light can be used to control a nano-switch, laying the groundwork for atomic-sized devices for use in a range of nanotechnologies. The fabrication of switches with nanoscale dimensions is a fundamental step forward in the further miniaturization of electronic devices, and the realization of fully-integrated nanotechnologies. Now, Dong Xiang and colleagues from Nankai University in China, working with fellow Chinese and international researchers, has developed a method that uses light to control the electrical conductance of the junction between gold nano-electrodes. By heating electrons at the surface of the electrodes with light, a technique known as plasmonic heating, the team was able to expand the electrodes and close the gap between them, turning the switch on. The work could pave the way for new nanotechnologies, including single-molecule transistors and nanopore-based biosensors.

## Introduction

Metallic quantum point contacts exhibit striking features, e.g., their atomic-scale dimension and electronic quantum transport, which have motivated extensive experimental and theoretical research in recent years^1–5^. Fabricating electronic devices with functional building blocks of atomic size is a major driving force of nanotechnology^6^. The electronic switches, which constitute a key element in electronic circuits, have been miniaturized to atomic scale^7–9^. Methods for the fabrication of atomic switches include mechanical tuning^1,10^, bias voltage/current operation^11–14^, and an electrochemical approach^11,15,16^. However, atomic switches controlled by plasmonic heating have not been discussed in previous studies. Coherent delocalized electron oscillations at the interface between two materials, which are known as surface plasmons (SPs), are capable of concentrating light into subwavelength gaps between two metallic nanostructures. When the resonance frequency of SPs matches the frequency of incident light, the plasmon resonance is excited, which produces strong light absorption and substantial plasmonic heating^17^. In this study, we show that a metallic atomic-scale contact can be reliably operated as a conductance switch by controlling the light illuminations.

The metallic atomic-scale contact was obtained utilizing the mechanically controllable break junction technique by precisely stretching a metal wire^18,19^. When the cross-section of a metal wire is reduced to few nanometers or a few atoms, the contact diameter becomes comparable to the Fermi wavelength of the electrons, then quantum-mechanical effects will strongly influence the electron transport properties^14^. In this study, we demonstrate that the conductance of an atomic gold contact can be toggled from few conductance quanta to hundreds of conductance quanta, and vice versa, using light illuminations, i.e., the conductance can be modulated between 1 *G*_0_ and 10^2^ *G*_0_, (*G*_0_ = 2*e*^2^/*h* = 77 μS), where *e* is the electron charge and *h* is Planck’s constant. We show that the metallic quantum contacts can be reversibly switched between the open state and the closed state by controlling the light intensity and switching the conductance between 10^−6^ *G*_0_ and 10^2^ *G*_0_. After the break of the quantum contacts (*G* < 1 *G*_0_), a nanogap is generated, in which coherent tunneling governs the electron transport.

The generation of a nanogap is crucial for the fabrication of single molecule-based devices. However, fabricating an adjustable atomic-scale gap to match the length of molecules in situ using standard nanotechnologies remains a significant challenge, although several advanced strategies (e.g., the electromigration method and the electrochemical atomic deposition approach) have been proposed to fabricate stable fixed atomic-scale gaps^20–23^. Typically, these fixed gap sizes cannot be adjusted as far as the fabrication process was finished. However, the gap size can be readily and continuously adjusted by applying the plasmonic heating to these fixed nano gaps. We demonstrated that the gap size can be modified with subangstrom resolution by controlling the light intensity or polarization. This precisely adjustable angstrom gap can be applied in a variety of research fields, such as the fabrication of single-molecule transistors, tip-enhanced Raman spectroscopy, and nanopore-based biosensors. Using various light sources and different substrate materials, combined with finite element method-based simulations, we demonstrate that the conductance modulations and gap size regulations are caused by the expansion of metal electrodes due to plasmonic heating.

## Results

### Atomic switching of metallic quantum contacts

**Fig. 1:**
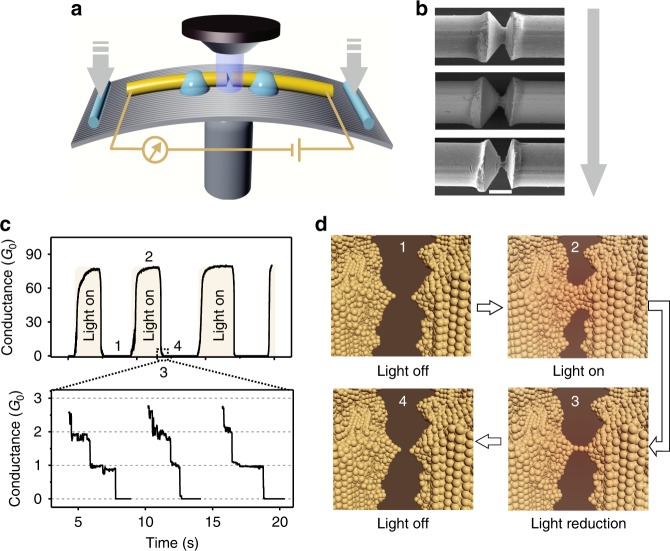
Strategy for the realization of atomic switching and conductance regulated by light illumination. **a** A metal wire with a notch in the middle is fixed on the substrate. The notch can be stretched until it finally breaks due to the bending of the substrate, which produces two separated electrodes. **b** SEM images of the notched microwire during the stretching process. Scale bar: 50 μm. **c** Real-time measurement of the current with the light switched on/off every 50 s–60 s. Zoomed image: conductance decreases in quantum steps at multiples of *G*_0_ (=2*e*^2^/*h*) as the light intensity decreases. **d** Schematic of the atomic arrangement, which corresponds to four conductance states upon light illumination. State 1: the two electrodes are separated by a few angstroms (*G* ≪ 1 *G*_0_). State 2: the two electrodes are reconnected upon light illumination (*G* ~ 80 *G*_0_). State 3: the two electrodes are stretched, and a gold atom chain is formed before the nanocontact breaks when the light intensity is reduced (*G* ~ 1 *G*_0_). State 4: the two electrodes are separated again due to the heat dissipation as the light is completely turned off (*G* ≪ 1 *G*_0_)

In our experiments, a commercial light-emitting diode (LED) lamp is employed as the light source with an AC adapter to continuously control the intensity of light (see Supplementary Figure S1). There is no need for special optical set-ups or high-power laser sources in the experiments. To fabricate the nanocontacts, a commercially available gold wire with a constriction in the middle is fixed on a spring steel substrate^19^. The constriction can be precisely stretched by bending the substrate using a mechanically controllable break junction (MCBJ) setup^24–27^, as shown in Fig. 1a. Figure 1b shows the scanning electron microscope (SEM) image of the gold metal wire during the mechanical stretching process. The image shows that the cross-section of the constriction is reduced until a final break upon bending of the substrate, which produces two separated electrodes (see Supplementary Figure S2).

At the initial stage, the gap between the two electrodes is set to a few angstroms (10^−5^ *G*_0_ < *G* < 10^−4^ *G*_0_) employing the MCBJ set-up. When the light is turned on, the conductance is increased from the value of *G* ~ 10^−5^ *G*_0_ to a stable value of *G* ~ 80 *G*_0_ with a 1–2 s delay following the light stimulation. When the light is turned off, the conductance decreases to a value far below 1 *G*_0_ with 1–2 s delay once again. This conductance switch behavior is reliably reproduced for each measurement period, as shown in Fig. 1c. The on/off switching ratio and the delay time are largely governed by the maximum value of the light intensity and the initial gap size between two electrodes. For our setup, a maximum conductance of 80 *G*_0_ is obtained when the light intensity reaches 1 mW/mm^2^ with an initial gap size ~1 nm. The large conductance (~80 *G*_0_) demonstrates that the two separated electrodes are strongly reconnected upon light illumination.

A closer examination of the conductance curve shows that the conductance rapidly decreases and reaches a region with plateaus in nearly all opening traces that occur at integer multiples of the conductance *G*_0_ = 2*e*^2^/*h*, as shown by the zoomed magnified area in Fig. 1c. This value is the well-known conductance quantum, which occurs when the size of a metallic contact is decreased to a single atom or a chain of gold atoms^28^. Note that we have a high chance (~90%) of obtaining these conductance plateaus, which indicates that the conductance of the system is controllable using the light control even at room temperature because of the fewer mechanical vibrations. As shown in Fig. 1c, each conductance step can last for several seconds, indicating that the separation of gold atoms (even separated one by one) is controllable using light illumination.

Figure 1d is a schematic of the atomic arrangement upon light illumination. At initial state 1, the two electrodes were separated by a distance of a few angstroms, and the corresponding conductance is substantially less than 1 *G*_0_. The nanogaps show a strong absorption of light in the visible and near-infrared regions due to their localized plasmon resonances, i.e., once the frequency components of the incident LED light match the oscillation frequency of the free electrons coupled with the electromagnetic field on the electrode tips, the localized surface plasmon resonance around the gap is excited. Note that both the quantum tunneling and nonlocal effects in a sharp gap are also involved^29,30^. The absorbed light is converted to thermal energy, leading to an expansion of the nanoelectrodes, and thus, the reconnection of the two nanoelectrodes. State 2 shows the reconnection of two electrodes upon light illumination, in which the corresponding conductance is near 10 *G*_0_–80 *G*_0_, depending on the light intensity. The conductance reaches its maximum value as the system is in thermal equilibrium. When the light intensity decreases, the metal wire is stretched due to the shrinkage of the electrodes, and an atomic point contact may be formed before the nanocontacts completely break, as shown in state 3. When the light is completely shut off, the two electrodes are separated again, and the electron transport returns to the tunneling regime (*G* ≪ 1 *G*_0_), as shown in state 4.

**Fig. 2:**
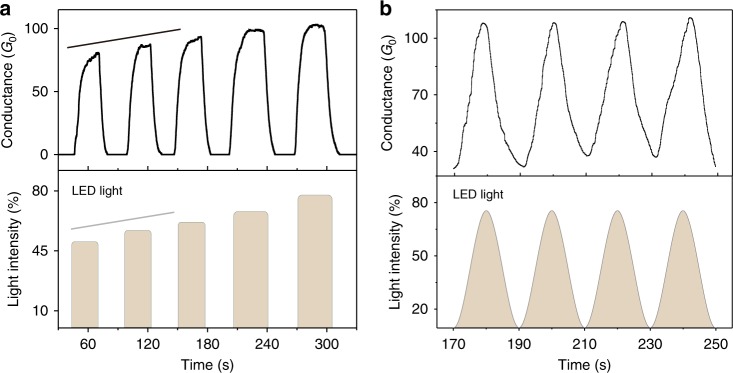
Conductance *vs* time as modified by the light intensity and light excitation form. **a** Bottom panel: The incident light is periodically switched on and off, while the maximum light intensity in each circle is gradually increased. Top panel: the conductance of the metallic contact increased following the trend of the light intensity. **b** The conductance of the metallic contacts changes similarly to a sinusoidal wave, following the sinusoidal light intensity waveform

To understand the dependence of conductance on the light intensity, we perform experiments where the maximum light intensity in each illumination circle is gradually increased. The maximum conductance in each circle increases approximately linearly with the light intensity, as shown in Fig. 2a. When the light intensity changes smoothly in a sinusoidal form, the conductance of the nanocontact follows the trend of the light intensity and sinusoidally varies, as shown in Fig. 2b. Additional repeatable data of the current as a function of the light intensity is observed in the Supplementary Video S1 and Figure S3. Based on these results, we conclude that the conductance of the quantum contact is regulated by the light intensity.

### Nanogap size precisely modulated by light

**Fig. 3:**
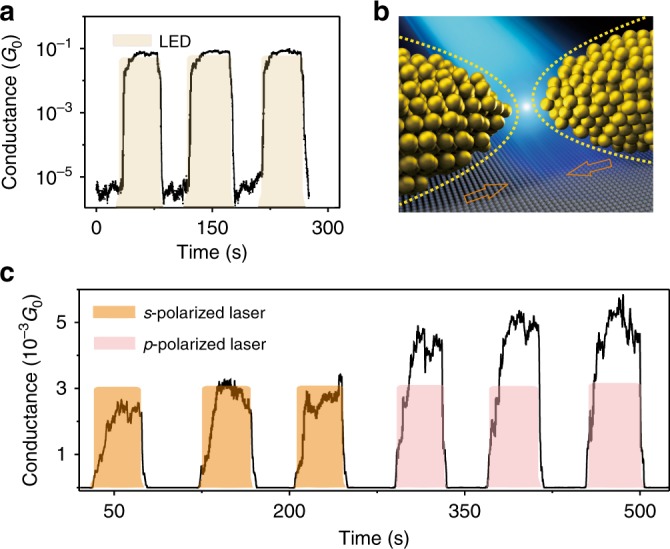
Dependence of conductance on the incident light. **a** Real-time measurement of the conductance upon the LED light illuminations in the tunneling regime. *V*_bias_ = 1 mV. **b** Schematic of the gap size variation upon light illumination. The dashed lines indicate the new position of the nanoelectrodes upon LED illumination. **c** The conductance of the tunneling gap dependent on the laser polarization. When a *p*-polarized laser (pink) is employed, the conductance is approximately two times larger than the conductance when an *s*-polarized laser (orange) is employed. The laser central wavelength is 640 nm with a bandwidth of 5.7 nm, and the maximum laser power density is 0.5 mW/mm^2^

After breaking the two electrodes (e.g., state 4 presented in Fig. 1d), we find that the gap size between the electrodes can be precisely modified by the light illumination. Figure 3a displays that the conductance can be modified in the tunneling regime [10^−5^ *G*_0_, 10^−1^ *G*_0_] by the control of LED light in ambient conditions. The tunneling current can be kept constant for a long time with a slight fluctuation if the light intensity is fixed. The distance between two electrodes can be estimated using the Simmons equation, which describes the relationship between the tunneling current and the tunneling gap size^31^. Our calculation shows that the gap size changes by only a few angstroms as the tunneling current changes from 10^−5^ *G*_0_ to 10^−1^ *G*_0_ (Supplementary Figure S4), which demonstrates that the distance between the two separated electrodes can be precisely controlled at subangstrom accuracy by the light intensity. Figure 3b shows a schematic of the gap size variation upon light illumination.

To further clarify the mechanism for the light-controlled tunneling current, we investigate the current response under laser irradiation, which possesses not only a small focus spot but also a specific polarization. First, we addressed the effect of the focus spot location on the tunneling current by recording the transient conductance when the laser focus spot is scanned along the long axis of the metal wire. The tunneling current is dramatically changed only when the laser is focused on the nano contact area, as shown in Supplementary Figure S5. Therefore, the nanogaps between the electrodes rather than other locations play key roles in the modification of the tunneling current. Second, we investigate the effect of laser polarization on the tunneling current. In our experiment, the electric field of the laser (~640 nm wavelength) can be adjusted to be parallel (*p*-polarization) or perpendicular (*s*-polarization) to the electrode axis with a focal spot of ~10 μm diameter on the nanocontact area. We observe that the conductance increases in the tunneling regime when the *s*-polarized laser illuminates the nanogap. Keeping the laser power at a constant value, a significant increase in the conductance occurs when the *s*-polarization laser is switched to *p*-polarization, as shown in Fig. 3c. The electric field is strongly enhanced and localized around the nanogap when the electric field of the incident light is parallel to the electrode axis (*p*-polarized laser), which will result in a strong absorption of the incident light^17,32,33^. The plasmonic heating is weaker when the electric field of the incident light is perpendicular to the electrode axis (*s*-polarized laser). Therefore, the dependence of the conductance on the laser polarization provides evidence that the gap size modulation is related to plasmonic heating.

### Expanding of nanoelectrodes induced by plasmonic heating

**Fig. 4:**
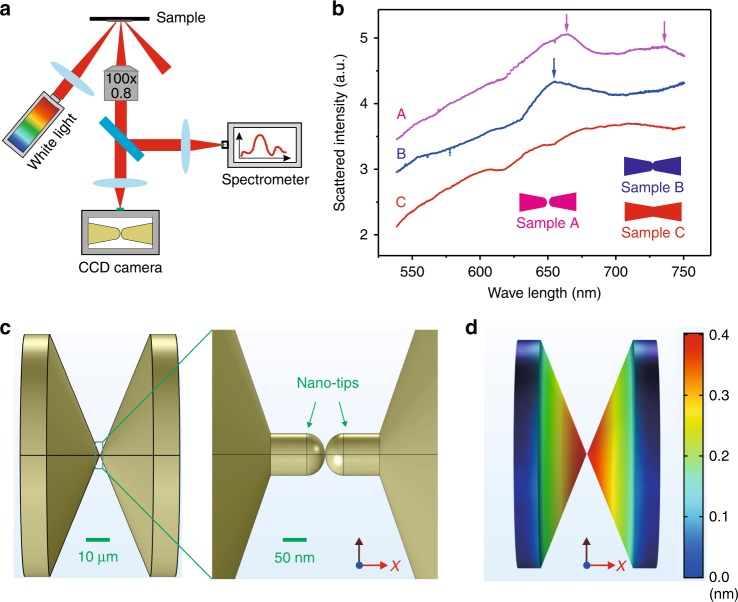
Characterization of MCBJ devices and simulation of expansion distribution of the electrodes upon light illumination. **a** System for the measurement of optical spectroscopy. **b** Measured dark field scattering spectra from the gap area that employs three different samples. The gap size is ~2 nm in sample A and ~0.2 nm in sample B. The electrodes were strongly reconnected, and no nanogap is observed in sample C. Plasmonic resonances are indicated by the arrows. **c** Model used in the simulation. Parts of the large metal wire close to the nanotips were considered. The gap size between two nanotips is initially set to 2 nm. The polarization of the incident light is parallel to the *x*-axis. **d** Expansion distribution (in X component) when equilibrium temperature was established

To confirm that the switching behavior originates from plasmon-induced heating in nanoscale plasmonic systems, the scattering spectrum of the MCBJ samples are investigated by which the frequency of plasmonic resonance is revealed. Figure 4a shows the measurement system, and more detailed information is provided in the Supplementary Information Figure S6. Figure 4b shows the measured dark field scattering spectra with different samples. The location of the plasmonic scattering peak weakly depends on the gap size and is centered near *λ* = 660 nm (refer to sample A and sample B). No scattering peak is observed in the regime from 400 to 600 nm for all samples. Subsequently, we perform the experiments of light modulated conductance with a 488 nm laser (power density equivalent to 640 nm wavelength), in which the main frequency detunes from the resonance frequency of the structure. The illumination of a 488 nm laser only produces a smaller change in conductance compared to the illumination of a 640 nm laser. The observation indicates that the conductance change is related to the expansion of the electrodes due to plasmonic heating.

We further perform simulations based on the finite element method, by which the expansion of the electrodes is estimated. Because the nanotips are directly connected to a macroscopic gold wire and the gold wire has excellent thermal conduction, the generated heating should not be localized on the nanotips. Thus, the part of the macroscopic wire between the two fixed points and the nanotip are considered in our model, as shown in Fig. 4c. Note that the thermal diffuse at the metal-substrate interface does not play an important role in determining the expansion of electrodes since the main part of the electrode tips are suspended above the substrate and the contact area between the cylindrical metal wire and flat substrate is very small. The nanoelectrodes are illuminated with a *p*-polarized plane wave at *λ* = 640 nm, for which the surface plasmon resonance can be excited, as shown in Supplementary Figures S6-S8. The electric field distribution, the temperature distribution, and the thermal expansion upon light illumination are solved using the COMSOL multiphysics program package^34,35^; see Supplementary Figures S9-S12 for detailed information.

Our simulation shows that the electric field intensity is substantially enhanced by ~10^3^ in the gap regime upon a *p*-polarized laser, as shown in Supplementary Figure S9. The heat density distribution can be obtained based on the electric field distribution. The heat power density is set as the heat source, and the thermal expansion/displace of the electrodes is simulated, as shown in Fig. 4d. The maximum displacement of the electrodes is ~0.4 nm. Considering two opposite electrodes, the gap size variation should be ~0.8 nm, which produces a large change in the tunneling current that exceeds several orders of magnitude.

**Fig. 5:**
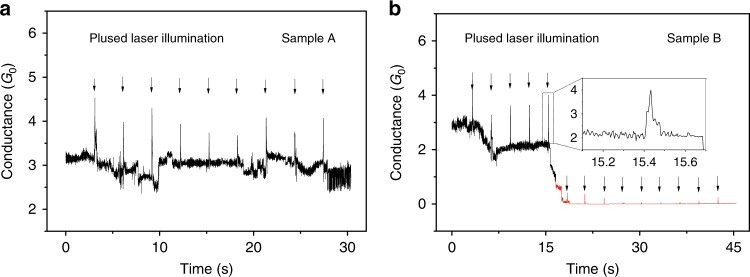
Test of current response time upon the light illumination with two different samples. A chopper was used to generate a pulse laser for the light illumination. The illumination time was set to 50 ms at every 3000 ms, and the arrows indicate that the junction was illuminated by a pulsed laser. **a** For sample A, the current of the quantum point contact switch between 3*G*_0_ and 4*G*_0_ upon the illumination of pulsed laser. **b** For sample B, the current of quantum point contact switch between 2*G*_0_ and 3*G*_0_ upon the light illumination. The zoomed figure shows that the time interval for the current change is ~60 ms upon the pulsed laser illumination. The red curve shows that the tunneling current can be quickly modulated by the pulsed laser after the separation of two quantum contacts

We perform additional experiments to probe the ultimate limit of switching time. It is found that the time for the current response (rising time or fall down time) can be reduced to a minimum of 10 ms by reducing the maximum intensity of the incident light since the expansion volume of the electrodes is positively related to the light intensity (Supplementary Figure S13). Similar to reducing the intensity of light, the reducing illumination time also produces a rapid response of current due to the lower thermal expansion. Assisted by a chopper, the illumination time of the laser is precisely reduced from seconds to milliseconds (Supplementary Figure S14). The total hold time for the high current is ~60 ms upon the illumination of the pulsed laser (illumination time is set to 50 ms), as shown in Fig. 5. The current response is only 10 ms delay upon the light illumination. This observation is consistent with the previous observation by reducing the light intensity, which confirms that the current switch can be quickly operated.

As an expectation, the switching frequency can be further optimized with a microfabricated sample, since the thermal diffusion time for the thermal expansion can be reduced to nanoseconds with a microfabricated sample based on the formula *L* = (*Dt*)^0.5^, where *D* is the thermal diffusivity and *L* is the length of the structure for heat diffusion^36^. We observe that the current change can follow the light intensity change even when the junction is illuminated by the laser at 50 Hz. However, it cannot be distinguished by the eyes when the light intensity changes at this frequency (refer to Supplementary Video S2 and Figure S15). This experiment provides further proof that the atomic can be fleetly operated via plasmonic heating.

## Discussion

The observation of conductance differences upon two light polarization states was small (a factor of two), which seems to disagree with the expectation of field enhancement theory (a factor of hundreds). This observation is attributed to the following reasons. (1) Although the electric field in the gap is strongly dependent on the light polarization, the absorption power has relatively weak dependence on the polarization direction, e.g., when the polarization of incident light is switched from perpendicular to parallel with respect to the interparticle axis, the electric field increases several hundred times, while the absorption power only increases by approximately one order of magnitude for 2 nm gaps^37^. The main reason is that the adsorption power is achieved from the whole structure, while the enhancement of the electric field normally refers to the maximum value at the hottest point. (2) The geometry of the cross-section of the broken gap is complex after the break of metal wire. The model that we employed for the simulation is an ideal geometry for a qualitative interpretation. Several pairs of tips have different orientations in the broken cross-section, i.e., a *p*-polarized light with respect to one pair of tips may be an *s*-polarized light for another pair of tips. Thus, a two-time relationship of the conductance upon *p*-polarization and *s*-polarization should be reasonable.

Other possible mechanisms exist for the conductance modulation upon light illumination. (1) One mechanism is the photothermoelectric effect on the conductance of the junctions, which arises when the temperature gradient, Δ*T*, across a junction is generated under light illumination^38,39^. This Δ*T* will generate a thermovoltage and an additional tunneling current. (2) Another mechanism is the effect of the plasmonic oscillating field on the conductance. In this mechanism, the plasmon field is treated as a potential, which oscillates at the plasmon frequency and promotes the transmission of an electron by broadening the electron energy level^39,40^. (3) A third mechanism is the effect of plasmon-induced hot electrons on the conductance^41,42^. The plasmon-induced hot electrons will enhance the tunneling current, especially as the light with a short wavelength was utilized. Although these effects may considerably contribute to the conductance modulation, the dominant mechanism for the conductance modulation should be the plasmon-induced heating. We obtain this conclusion based on the following facts: (1) the conductance of the nanojunction can be modulated across eight orders of magnitude from 10^−6^ *G*_0_ (tunneling transport) to 10^2^ *G*_0_ (ballistic transport), which suggests that the metal wire is broken and reconnected upon light illumination; (2) a delay time between the maximum of the light intensity and the maximum of the conductance indicates that the conductance modulation is caused by the gradually expanding nanoelectrodes until an equilibrium of heating dissipation and heating generation is established; (3) the observation of the conductance dependent on laser polarization and laser frequency shows that surface plasmon resonance is significantly attributed to the modulation of the tunneling current; and (4) our simulations further justify that a relative large-scale expansion of the nanoelectrodes can be induced by plasmonic heating.

We note that although a prerequisite nanogap is needed to perform the gap size modulation by plasmonic heating in our experiments, this prerequisite nanogap can also be generated by other fabrication techniques, such as electromigration, chemical deposition, shade evaporation, and a dash-in-line lithography technique^6^. With these on-chip fabrication techniques, a highly integrated nanogap array may be fabricated, which enables the realization of adjustable on-chip nanogaps controlled by light, which is unavailable for the MCBJ technique that is solely driven by piezo ceramics. We propose a new strategy for the precise control of the gap size by plasmonic heating, which has the potential to be independent of the MCBJ technique.

In contrast to previous reports, in which the switches behavior was triggered by the intrinsic molecular conformation transition upon external light, we demonstrated that a conductance switch can be realized by bare electrodes without any molecules. We demonstrated that the atomic geometry of metallic quantum contacts can be considerably modulated with incident light, and the conductance of quantum contacts can be reversibly switched between 10^−5^ *G*_0_ and 10^2^ *G*_0_ by resonant plasmonic heating. The one-atom by one-atom separation of two electrodes can be clearly observed, and the gap size between two electrodes can be continuously adjusted at a subangstrom resolution by the control of light intensity. The plasmon has the ability to break through the diffraction limit of light to realize nanofocusing; thus, the plasmon-controlled atomic switch may pave the way for the realization of highly integrated devices.

## Materials and methods

The evolution of the constriction upon stretching was monitored by measuring the junction conductance. The voltage applied across the metal wire is 1 mV, and the experiments are performed in ambient condition. Once the constriction of the metal wire breaks upon the stretching force, the electron transport enters the tunneling regime (*G* ≪ 1 *G*_0_). On the contrast, the conductance value *G* is big than 1 *G*_0_ when the two separated electrodes was reconnected (point contacts status).

## Supplementary information

Supplementary Information

Current modulated by the light illumination

Laser illumination system with different frequencies
