# Supplementary material for: Atomic switches of metallic point contacts by plasmonic heating
Source: Light Sci Appl. 2019 Mar 27;8:34. doi: 10.1038/s41377-019-0144-z (PMC6437168; doi:10.1038/s41377-019-0144-z)
Supplement: Supplementary file 1 — Supplementary Information [file 41377_2019_144_MOESM1_ESM.docx]

**Supplementary Information for**

**Atomic switches of metallic point contacts by plasmonic heating**

*Weiqiang Zhang^1^, Hongshuang Liu^1^, Jinsheng Lu^2^, Lifa Ni^1^, Haitao Liu^1^, Qiang Li^2^, Min Qiu^2^, Bingqian Xu ^1,3*^, Takhee Lee^4*^, Zhikai Zhao^1^, Xianghui Wang^1^, Maoning Wang ^1^, Tao Wang^5^, Andreas Offenhäusser^6^, Dirk Mayer^6*^, Wang-Taek Hwang^4^ and Dong Xiang^1*^*

^1^Tianjin Key Laboratory of Optoelectronic Sensor and Sensing Network Technology, Key Laboratory of Optical Information Science and Technology, Institute of Modern Optics, College of Electronic Information and Optical Engineering, Nankai University, Tianjin 300071, China

^2^State Key Laboratory of Modern Optical Instrumentation, Department of Optical Engineering, Zhejiang University, 310027, Hangzhou, China

^3^College of Engineering, University of Georgia, Athens, Georgia 30602, USA

^4^Department of Physics and Astronomy, and Institute of Applied Physics,Seoul National University, Seoul 08826, Korea

^5^Institute of Materials Research and Engineering, A*STAR, 2 Fusionopolis way, Innovis, Singapore 138634, Singapore

^6^Institute of Complex Systems, ICS-8. Bioelectronics, Research Center Juelich and JARA Fundamentals of Future Information Technology, Jülich 52425, Germany

**1. Fabrication of the nanocontact**

In this letter, mechanically controllable break junction (MCBJ) technique is used to fabricate the initial metal point contacts (MPCs) of gold in ambient conditions. The optical system is shown in Figure S1a and the MCBJ setup is shown in Figure S1b. A gold wire with a diameter of 100 μm is fixed on the MCBJ substrate with a cyanoacrylate adhesive. Spring steels sheets (length of 40 mm, a width of 12 mm and thickness of 0.2 mm) were used as the substrates. A flexible isolating layer consisting of polyimide (HD-4100, HD Microsystem, and ~2 μm thick) was spin coated onto the spring substrate. To complete the imidization of the polyimide, the substrate was baked in an oven at 100 ^o^C for half an hour, and a second layer of polyimide was spun on the substrate in order to reduce the probability of shorts and defects.

**Fig. S1 | Measurement system**. **a**, The test platform for the polarized laser illumination and electrical characterization, which includes a probe station, optics parts, a mechanically controllable break junction setup, and current measurements devices (not shown). **b**, A homemade MCBJ setup with a bent substrate. The two outer posts of the three-point bending apparatus were fixed, and the third post (push rod) can move in the vertical direction to bend the substrate (chip).

To ensure that the metal wire break easily, a notch was made with a knife in the middle of the wire. Controlling the movement of the supporting platform in the X direction and the force exerted on the knife in the Z direction allowed the gold wire to be mechanically roundly cut down without complete breakage, see Figure S2a. The optical image and the SEM images of the notch part are shown in Figure S2b-2d.

**Fig. S2  |  The fabrication of nanocontact**. **a**, Setup to roundly cut the metal wire. The metal wire was sandwiched between a knife blade and a supporting platform. The platform can move in the vertical (Z) and parallel (X) directions with a resolution of ~5 μm. **b**, SEM image of the nanogap after breakage of nanocontact. Scale bar: 5 μm. **c**, Optical micrograph of the metal wire with a notch in the middle. Scale bar: 50 μm **(d)** SEM image of the metal wire. Scale bar: 20 μm.

The metal wire can be precisely stretched by bending the substrate using mechanically controllable break junction (MCBJ) technique. The main part of the MCBJ setup was fabricated with steel to suppress the inelastic deformation. The maximum dimension of the system was 80 mm × 50 mm × 40 mm. A piezo was chosen as a push rod which can move in the vertical Z direction controlled by the piezo actuator. The cross-section of the notch will be reduced, and metal point contacts can be generated when the substrate is bent by the control of push rod.

**2. Electrical characterization of the nanocontact**

All the current-voltage responses and current-time responses were recorded real-time with the semiconductor device analyzer (B1500A) from Keysight Technologies at an interval of 4 ms. For the measurement of the small tunneling current, the ASU (atto sense/switch unit) and integrated amplifiers are added to promote the measurement sensitivity, *i.e*., the detected lowest current is below 10 pA. To precisely control the movement of the push rod, an ultra-precise nanopositioning piezoceramic (a step size of 10 nm) from ATTOCUBE is used. Taking the attenuation factor (defined as: r = ΔX/ΔZ, where, ΔX is the gap size change between the two nanoelectrodes, and ΔZ is the displacement of the push rod. For this sample, and r ~10^-3^ can be reached) into consideration, the elongation of the suspended notched can be controlled with an accuracy of 0.1 Å. Additionally, a black metal shielding box and an optical platform are used to reduce the noise from external electromagnetic waves and mechanical vibration.

**Fig. S3  |  Conductance *vs* time as modified by the light intensity.** The conductance of the metallic contacts changes similar to a triangular wave, following the triangular light intensity waveform. The current control by illumination of light can be reproduced hundreds of times. The maximum intensity of the light and period in each circle is not equal, which lead to the fluctuation of the maximum of current. “On” represents the state that the two gold electrodes are directly contacted (G > 1G_0_), and “off” indicates the state that the two gold electrodes are separated.

**3. The Estimation of gap size**

The distance between two electrodes can be estimated based on the Simmons equation, which describes the relationship between tunneling current and tunneling gap size^S1^. Figure S4 shows the calculated relationship between gap size and the tunneling current, in which two tunneling barrier values were used to determine the gap size. An electron tunneling barrier *ø* = 4.0 eV is normally used for a vacuum condition (*ø* dependent on the electrode material), and *ø* = 1.0 eV is typically used for a solution condition^S1^. The tunneling barrier in our experiment (in air) should fall in between these two regimes (1.0 eV, 4.0 eV). Figure S4 shows that the gap size changes only few angstroms as the tunneling current changed from 10^-6^ G_0_ to 10^-1^ G_0_, manifesting that the distance between the two separated electrodes can be precisely controlled at sub-angstrom accuracy by the light intensity.

**Fig. S4  |  The calculated relationship between the tunneling current and gap size.** The red and blue curves were calculated using tunneling barrier *ø* = 4 eV and *ø* = 1 eV, respectively.

**4. LED light and laser irradiation**

To perform the light irradiation experiment, a white LED with a power of 0−20 mW was placed on top of the MCBJ at a distance of ~5 cm. In addition, a laser (0−120 mW, 640 nm) from LASERWAVE Optoelectronics Technology Co. Ltd. is used for further verification. The output power of both light sources can be adjusted continuously. We found that the conductance shows less fluctuation over a few minutes as the light intensity is fixed.

Figure S5 shows the transient conductance change when the laser spot (~10 μm in diameter) was scanned along the long axis of the sample with a power of 10 mW. We found that an enhancement of the conductance appeared only when the light was focused on the nanocontact part. Additionally, we found that no conductance change was observed when a heat source (such as a bottle of 90 ^o^C water, 1 mm above the sample) was scanned along the axis of the metal wire. These observations indicate that the thermal expansion of other parts can be ignored., *i.e.*, only the nanocontact part plays a dominant role in conductance modulation.

**Fig. S5  |**   A sketch of laser-induced conductance change (ΔG) during laser scanning along the long axis of the electrodes. The conductance dramatically changed when the laser was focused on the nano-contact part.

In addition to the semiconductor laser, we found that the conductance of nanocontact can also be modulated with a light-emitting diode (LED, RS72). The spectra of the two types of sources are shown in Figure S7. It can be found that the intensity is located between 450 nm−650 nm, and the light in these regimes can be strongly absorbed by the plasmonic system and converted to thermal energy due to the Ohm loss.

**5. The measurement of optical spectra**

We performed the measurement of dark-field (DF) scattering spectra using three types of MCBJ samples. The optical setup for the DF scattering measurement is shown in Figure S6a. We use an oblique incidence white light (wavelength 400−1000 nm) with an incident angle of 70 degrees as the excitation source (Thorlabs, OSL2). Most white light can be reflected or be refracted by the sample according to the principle of light reflection or refraction, while the light scattered by the nanogaps can be collected with a high numerical aperture microscopic objective (Olympus, 100× magnification, NA = 0.8), which enable DF spectra with excellent signal-to-noise levels. The white light source is focused with a lens to make sure the radius of the light spot is smallest on the junction of the sample, and then the light scattered from the sample is collected by the microscopic objective. The scattered light is split by a beams splitter (BS), with 80% of the light focused into a spectrometer (Andor, SR500i) and the other 20% passing to a CCD camera for imaging. By adjusting the size of the slit and selecting the pixels of the zero-order imaging in the spectrometer, the same effect as the pinhole imaging can be achieved, ensuring that the collected scattered light is completely from the nanostructures in the junction. Spectrometer integration times of 30 ms were used and measured spectra were normalized to the scattering of a whiteboard replaced at the sample position. In addition, in order to eliminate the interference of the substrate material on the scattered light, we have specifically selected a substrate, which is transparent (polyvinyl chloride) to visible light, for the measurement of scattering spectrum.

Figure S6b shows the measured scattering spectrum from the nanogap area. It can be found that the plasmonic resonances frequencies were mainly located around 650 nm. The resonance peaks depend on the gap size and the shape of electrodes, which agrees well with the simulated results as shown in Figure S8. We further performed the experiments with a 488 nm laser detuned from the resonance frequency. Unlike the laser diode light source (semiconductor laser, LWRL635, 640 nm) and light-emitting diode (LED, RS72), the illumination of 488 nm only results in a smaller changes in conductance.

**Fig. S6  |  The measurement of** **dark-field scattering. a,** The system for the measurement of optical spectroscopy. **b,** Measured dark field scattering spectra from the gap area employing three different samples. The gap size is ~ 2 nm in sample A. The two gold electrodes were weakly reconnected (~ 0.2 nm in nanogap, sample B) and strongly reconnected (no nanogap, sample C). Plasmonic resonances are indicated by the arrows.

**Fig. S7  |  The spectroscopy of the incident light source.** (a) LD light source (semiconductor laser, LWRL635) and (b) light-emitting diode (LED, RS72).

**6.  Simulation of electromagnetic scattering and thermal expansion**

To find the plasmon resonant frequency, the heat absorption spectra of the nano structures were studied. For convenience, only one pair of nanoelectrodes (radius of the spheroidal part *r* = 30 nm, radius of cylindrical part *R* = 30 nm, and length of cylindrical part *L*= 60 nm) was built, see Figure S8a. The gap size between two symmetrical electrodes is set to be 0.5−2 nm. At the frequency range of visible light (400 nm − 700 nm), the gold can be modeled as having a complex permittivity with both real and imaginary components, which can be extracted from the literature^S2^*.* The incident plane wave propagated along the X direction, and the electric field (*E*) polarized along the Z-axis (*s*-polarization) or Y-axis (*p*-polarization), see Figure S9a. The default boundary condition is perfect electric conductor, which applies to all exterior boundaries. We choose a spherical surface as the boundary condition with two layers. The outermost layer represents the PML (perfectly matched layer) acting as an absorber of the scattered light, and the second layer is the surface of the inner air domain. The thickness of both layers is set to be 300 nm, which is close to the value of λ/2. The incident electric field is set to 868·EXP (-j·ewfd·k_0_·X), where the maximum amplitude corresponds to a light intensity of 1 mW/mm^2^.

To calculate the heat generation within the nanoelectrodes system, the electromagnetic scattering of the nanostructure was first solved. Having the knowledge of the incident field and nanostructure composition, we can numerically solve the electromagnetic (EM) scattering, which is done with the commercial FEM solver in COMSOL Multiphysics (Electromagnetic Waves, Frequency Domain). We found that the position of the resonance peak shifted when the gap size and the polarization direction were changed. We noticed that the output electrical field for the *p*-polarized laser is much higher than for the *s*-polarized laser under the same intensity of incident light. Based on the distribution of the electrical field, the distribution of the temperature can be obtained, see the next section for a detailed explanation. Subsequently, a variable for the total heat loss in the gold was added as a volume integral of the resistive losses. In this way, the absorption spectra of light can be generated, as shown in Figure S8b. It can be found that the resonance frequency is strongly dependent on the gap size and laser polarization, and the wavelength used in our experiments (λ = 640 nm) falls in the range of the plasmon resonance frequencies.

**Fig. S8  |  Spectra of the absorption power**. **a**, The schematic illustration of the incident light impinging on a pair of nanoelectrodes. **b**, The absorbed power of light *vs* the wavelength for the gold model with different gap sizes and two incident polarizations.

Figure S9 depicts the simulation results of the electric field distribution upon incident light with different polarization (*p*-polarization and *s*-polarization). We found that the electric field was enhanced in both cases, but *p*-polarized laser showed much stronger enhancement than the *s*-polarized laser. Thus, the dominant effect of the local surface plasmon resonance is further verified by the simulation results. To study the thermal expansion of the nanoelectrodes upon laser illumination, three coupled modules including the “Frequency Domain Wave Electromagnetics”, “heat transfer in solids”, and “solid mechanics” were used with the COMSOL Multiphysics software. Here, we only studied the stationary process during the illumination of a vertical-incidence plane wave. The refractive index n_Au_= -2.45 -3.7i at the illumination wavelength of 640 nm is used in our model. The convective heat transfer coefficient is set to be 300 W/(m^2^× K) for nanotip and 5 W/(m^2^ × K) for big connected wire according to Ref. S7 and the radiation was neglected in the module of “heat transfer in solids”. For the gold nanoelectrodes, the maximum element size is set to be 5 nm, which is much smaller than the λ/2 value of the incident light. The intensity of the incident light is 1 mW/mm^2^ corresponding to a power of 20 mW.

**Fig. S9  |  Electric field distribution for the (a) *s*-polarized laser and (b) *p*-polarized laser upon irradiation.** For the *s*-polarized laser, the electric field is perpendicular to the axis of paired electrodes. For *p*-polarized laser, the electric field is parallel to the axis of paired electrodes.

The heat power volume density Q_d_ can be written as^S3^

$Q_{d}=\frac{1}{2}\varepsilon_{0}\omega\mathrm{Im}(\varepsilon_{\mathrm{r}})\left|\mathbf{E}\right|^{2}$ (1)

where *ε*_0_ is the permittivity of vacuum; *ω* is the angular frequency of light; *ε*_r_ is the relative permittivity of gold; and **E** is the electric field. From this formula, we know that the source of heat is locally proportional to the electric field intensity and the imaginary part of the dielectric function *ε*_0_^S4^. Once we know the intensity of the enhanced electric field, we can obtain the heat density distribution Q_d_. Subsequently, the power density *Q_d_* is set as the heat source for the coupling module “thermal expansion” in the multiphysics software. For convenience, the steady thermal diffusion equation can be reduced to^S5, S6^

(2)

where *T*(**r**) is the distribution of temperature produced by the external heat source *Q_d_* and κ(**r**) is the thermal conductivity. We can get the temperature distribution from this formula. The thermal expansion coefficient of the Au in our simulation is set to be 4.2 × 10^-5^ (1/K). We fixed both ends of the nano-electrodes by added constraint condition in the model to match the experimental condition. The initial temperature of the gold particles is set to 300 K, corresponding to laboratory temperature.

Due to the fact that the nanotips are directly connected to a large wire and the gold wire has excellent thermal conduction, the large wire between the two fixed points was taken into consideration for thermal expansion, see Figure S10. The parameters of the two tips are the same as those in the main text (100 µm in diameter and 50 µm in length). The gap between the two tips is set to be 2 nm. In the simulation, the light intensity is changing with time in the form of a square wave, and the heat power density can then be described by a rectangle function as Q(x,y,z,t) = Q(x,y,z) rect((t-t_0_)/τ). The temperature T(t) can be calculated using the heat conduction equation: $C\partial T\left(r,t\right)/\partial t+\nabla\cdot\left(k\nabla T\left(r,t\right)\right)=Q_{d}\left(r,t\right)$, where *C* and *k* are the thermal capacity and thermal conductivity, respectively. Coupled with solid mechanics module, the displacement or expansion of the structure can be obtained.

**Fig. S10  |  Structure used for the simulation**. **a**, schematically drawing of the samples. The distance between two fixed points is approximately 0.2 mm. **b**, Model used in the simulation (Whole electrodes model). The whole metal structure including the big gold wire between two fixed points and the nanogap were taken into consideration. The gap size is set to be 2 nm.

As shown in Figure S11a-b, the temperature of the structure (the point at the end of the microelectrode) changes slowly with a time delay of few seconds although the light intensity or heat power density changes fast (with a rising and falling time of 0.05 s) if the large wire (whole electrode model) was taken into consideration. The expansion would change synchronously with the temperature since the expansion is directly related to the temperature change on the equation δx = α × δT × L (here, α is the thermal expansion coefficient). On the contrary, the temperature of the structure changes with a time delay of only few milli-seconds follow the change of light intensity if only nano-tip model was taken into consideration, see Figure S11c-d.

**Fig. S11  |  Simulation results with the micro- or nano- structure employing COMSOL.** **a**, The transient intensity of light as a function of time. **b**, The temperature changes at the end of the micro- structure upon the illumination of light (as shown in Fig. S11a). **c**, The transient intensity of light as a function of time. **d,** The temperature changes when only nano-tips were taken into consideration upon the illumination of light (as shown in Fig. S11c).

Figure S12a shows that the temperature of the whole structure is uniform at t = 5 s, which indicates that the whole structure is heated to an equilibrium temperature at the time scale of seconds even though the heat power is mainly localized at the two tips (the electromagnetic field is mainly localized at the two tips). Figure S12b shows that the distribution of the thermal expansion. It can be found that the expansion of the tip can be up to 8 Angstroms. Thus, the increase in conductance occurs with a time lag of a few seconds upon the light illumination is reasonable when the big part of the structure connected to the nanotip is considered.

**Fig. S12  |  The simulation of temperature and expansion distribution of the electrodes upon light illumination. a,** The temperature distribution of the whole structure when *t* = 5 s, in which an equilibrium temperature was established. The initial temperature before the light illumination is set to be 300 K. **b**, Expansion distribution in X component when *t* = 5 s.

**7. Probe the limit of current response time upon the light illumination**

We found that the switch time can be reduced to 10 ms just by reducing the maximum intensity of incident light. As shown in Figure S13, the response time for the current is approximate 10 ms upon the illumination of light (~ 0.5 mW). Notably, ~10 ms is also needed for the switch of light source, which means the response of current can follow the change of light intensity in real-time. Also, 10 ms is already close to the sampling limitation of our measurement system for the recording of extremely low current.

**Fig. S13  | The switch of tunneling current upon the light illumination.** Upon the light illumination, the tunneling current is increased to dozen pA with a rise time ~10 ms. The gray shadow indicates that the real current is below the detection limit of the measurement device, and the tunneling current in this regime is strongly affected by the electric capacity due to the variation of gap size.


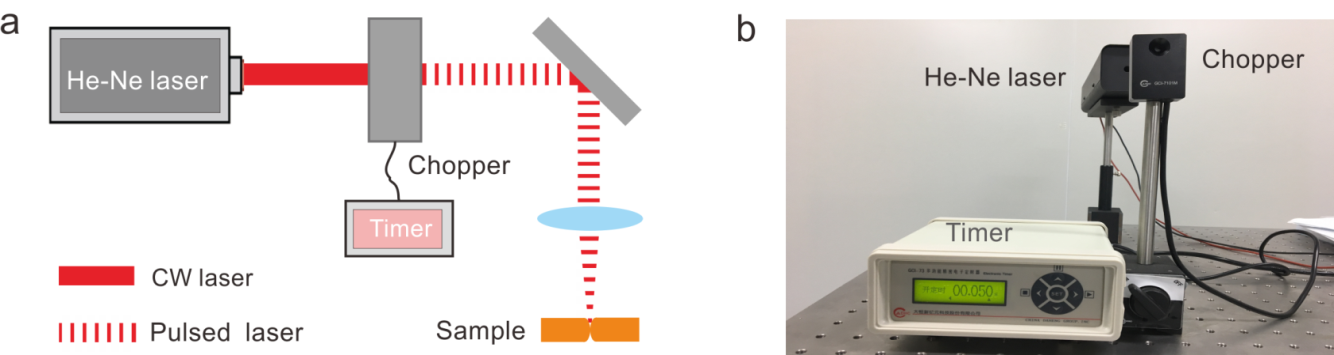


**Fig. S14 | Pulse laser experiment system using timer and chopper. a,** Schematic of optical setup for continuous laser (CW) conversion to pulsed laser. The duty cycle can be adjusted by the timer, and the highest resolution is 1ms. **b**, The real pulsed laser illumination system which includes a light source (semiconductor laser, LWRL635), a chopper (GCI-7101M) and a timer ( GCI-73).

Besides, we observed that the switching frequency can be further optimized with a micro-fabricated sample (see Figure S15), since the thermal diffusion time for the thermal expansion can be reduced to nanosecond with a micro-fabricated sample based on the formula L= (Dt)^0.5^, where, D is thermal diffusivity and L is the length of the structure for heat diffusion.

**Fig. S15 | Conductance change of the junction upon the light illumination with different frequencies.** **a**, The current response of a micro-fabricated junction when it is illuminated by laser with different frequency. **b**, Schematic of laser illumination system. The frequency of illumination laser is controlled by the current via a function generator and the current is amplified by a buffer. **c**, The real laser illumination system which includes a light source (650 nm, HT-30), a buffer (BUF 634), a synthesized function generator (DS 345), and a stabilized voltage supply (LPS 305).

**References**

S1 Grüter, L., González, M. T., Huber, R., Calame, M. & Schönenberger, C. Electrical conductance of atomic contacts in liquid environments. *Small* 1, 1067-1070 (2005).

S2  Johnson, P. B., & Christy, R.W. Optical constants of the noble metals, *Phys. Rev. B* **6**, 4370−4379 (1972).

S3 Chen, X. Y., Chen, T., Yan, M., & Qiu, M. Nanosecond photothermal effects in plasmonic nanostructures. *Acs Nano* **6**, 2550-2557 (2012).

S4 Govorov, A. O., & Richardson, H. H. Generating heat with metal nanoparticles. *Nano Today* **2**, 30-38 (2007).

S5 Richardson, H. H., Carlson, M. T., Tandler, P. J., Hernandez, P., & Govorov, A. O. Experimental and theoretical studies of light-to-heat conversion and collective heating effects in metal nanoparticle solutions. *Nano Lett.* **9**, 1139-1146 (2009).

S6 Baffou, G., Quidant, R., Girard, C. Heat generation in plasmonic nanostructures: Influence of morphology. *Appl. Phys. Lett.* **94**, 153109 (2009).
